# PsGRASP, a Golgi Reassembly Stacking Protein in *Phytophthora sojae*, Is Required for Mycelial Growth, Stress Responses, and Plant Infection

**DOI:** 10.3389/fmicb.2021.702632

**Published:** 2021-07-08

**Authors:** Jierui Si, Yong Pei, Peiyun Ji, Xiong Zhang, Ruofei Xu, Huijun Qiao, Danyu Shen, Hao Peng, Daolong Dou

**Affiliations:** ^1^Department of Plant Pathology, Nanjing Agricultural University, Nanjing, China; ^2^Department of Crop and Soil Sciences, Washington State University, Pullman, WA, United States

**Keywords:** *Phytophthora*, GRASP, mycelial growth, ER stress, pathogenicity

## Abstract

Golgi reassembly stacking proteins (GRASPs) play important roles in Golgi structure formation, ER stress response, and unconventional secretion in eukaryotic cells. However, GRASP functions in oomycetes haven’t been adequately characterized. Here, we report the identification and functional analysis of *PsGRASP*, a GRASP-encoding gene from the soybean-infecting oomycete *Phytophthora sojae*. Transcriptional profiling showed that *PsGRASP* expression is up-regulated at the infection stages. *PsGRASP* knockout mutants were created using the CRISPR/Cas9 system. These mutants exhibited impaired vegetative growth, zoospore release and virulence. PsGRASP was involved ER stress responses and altered laccase activity. Our work suggests that PsGRASP is crucial for *P. sojae* development and pathogenicity.

## Introduction

Oomycetes are fungus-like eukaryotic microorganisms belonging to the Stramenopile kingdom. However, they are evolutionarily distant from fungi but closely related to photosynthetic algae such as the brown algae and diatoms. Hence, most fungicides are not effective for oomycete control (Tyler et al., 2006; Gisi and Sierotzki, 2015). Oomycetes within the genus *Phytophthora* constitute a large group of destructive plant pathogens. For example, *Phytophthora sojae* causes soybean damping-off and root/stem rot, with annual losses estimated at $1–2 billion worldwide (Tyler et al., 2006).

*Phytophthora* pathogens utilize the asexual zoospores and the sexual oospores to penetrate host defense and achieve pathogenic success (Judelson and Blanco, 2005). *Phytophthora* oospores can survive in harsh environments, while sporangium-derived zoospores are infectious. In flooded soil, zoospores are formed in cytoplasm-cleaved and nucleus-separated sporangia (Judelson and Blanco, 2005). They swim chemotactically to host, and then encyst and form germ tubes to penetrate the host surface. Infection hyphae grow in the intercellular space of the host cells with secreted effectors delivered into the apoplast and the cytoplasm.

Recent evidence suggests that *Phytophthora* effectors are discharged from the haustoria through at least two mechanisms. Secretion of the EPIC1 protease inhibitor can be blocked by brefeldin A (BFA), indicating that it is delivered via the classic Golgi-mediated pathway. In contrast, delivery of the cytoplasmic RxLR effector Pi04314 is BFA insensitive despite that it contains a classic signal peptide (SP), suggesting the existence of an alternative route (Wang et al., 2018). Meanwhile, *Phytophthora* isochorismate (PsIsc1) which disrupts the plant salicylate metabolism pathway by suppressing its precursor may undergo the unconventional secretion pathway due to the lack of SP (Liu et al., 2014). Nonetheless, the modes and mechanisms of *Phytophthora* effector secretion are not clear.

As part of the endomembrane system, the Golgi complex receives transport cargos from the endoplasmic reticulum (ER), and then sorts and targets them to their final destinations. Functional Golgi needs to be correctly assembled as a flattened membrane sandwich, which is kept together by a protein matrix. The Golgi reassembly stacking proteins (GRASPs) play an important role for Golgi structure formation and have been identified in all eukaryotic cells except plants, where the *GRASP* genes are lost (Vinke et al., 2011; Rabouille and Linstedt, 2016). GRASPs are primarily found localized to the cytoplasmic surface of Golgi membranes, but their distribution into other cellular compartments is also expected (Zhang and Wang, 2020). Impaired Golgi stack has been observed in numerous *GRASP* knock-out or knock-down mutants (Tang et al., 2010; Bekier et al., 2017). Recent studies implicate GRASPs are also involved in autophagy, unconventional secretion, and other cellular activities such as growth, cell adhesion and migration in mammalian (Zhang and Wang, 2016, 2020). Interestingly, mammalian cells harbor two GRASP isoforms (55 and 65) whereas only one copy was found in lower eukaryotes, such as yeast and parasites (Behnia et al., 2007; Yelinek et al., 2009; Vinke et al., 2011).

In mammalian cells, GRASP55 and GRASP65 play complementary roles in maintaining the Golgi structure by forming *trans*-oligomers. Depletion of both impairs Golgi functions in protein trafficking, sorting and glycosylation, and reduces cell adhesion and migration. GRASP55 also participates in unconventional protein secretion of both cytosolic and transmembrane proteins in response to certain stress stimulus. The newly uncovered roles of GRASP55 as an energy sensor in the Golgi and a membrane tether in autophagy indicate that it may serve as a stress sensor and an effector in stress response. These roles may be linked to unconventional secretion of certain cargo molecules (van Ziel et al., 2019). GRASP55 expression is induced by the unfolded protein response (UPR) activated by ER stress inducers (Shemorry et al., 2019), which may affect GRASP55-mediated unconventional secretion.

GRASP-mediated unconventional secretion has been reported in multiple organisms including *Dictyostelium discoideum, Saccharomyces cerevisiae, Pichia pastoris*, and *Drosophila melanogaster* (Kinseth et al., 2007; Duran et al., 2010; Manjithaya et al., 2010). These events are not constitutive but stress-induced (Giuliani et al., 2011). For example, unconventional trafficking of the ER-retained mutant cystic fibrosis transmembrane conductance regulator (CFTR) can be triggered by ER stress-inducing treatments and GRASP55 overexpression in mammalian cell lines (Gee et al., 2011). The single GRASP ortholog (GrpA) in *D. discoideum* is required for unconventional secretion of acyl-coenzyme A-binding protein (AcbA) during spore differentiation (Kinseth et al., 2007). In *Cryptococcus neoformans*, GRASP is a key regulator of RNA vesicular export (Peres da Silva et al., 2018), and required for normal polysaccharide secretion and microbial virulence (Kmetzsch et al., 2011).

Likewise, oomycete GRASPs may play an important role in Golgi-mediated effector secretion. However, very little attention has been paid to GRASPs in filamentous pathogens including oomycetes. Here, we predicted a single GRASP protein from *P. sojae* (PsGRASP) and performed functional characterizations. *PsGRASP* showed highly induced expression at the infection stages. Knockout of *PsGRASP* led to defects in growth, zoospore release, and response to ER stress as well as a reduction in virulence. Our study suggests an essential role of GRASPs in oomycete development and pathogenicity.

## Materials and Methods

### Bioinformatics Analysis

To predict GRASPs in each genome, the hidden Markov model (HMM) profile of GRASP55_65 (PF04495) was obtained from PFAM database, and the HMM profile was used to perform HMM search against proteins from each genome using HMMER software. The GRASPs proteins were aligned using maximum-likelihood method of MEGA5 with the default parameters to investigate the relationships of GRASPs in distinct eukaryotic organisms.

The secretion signal of putative laccase proteins was predicted using SignalP 3.0^1^. The unconventional protein secretion of putative laccase proteins were predicted using SecretomeP 2.0^2^.

### *P. sojae* Strains and Culture Conditions

*Phytophthora sojae* strain P6497 used in this work was obtained from Professor Brett Tyler (Oregon State University, United States). The strain was cultured on 10% V8 media at 25°C in the dark (Erwin and Ribeiro, 1996). For stress response assays, the V8 agar medium was supplemented with 5 mM H_2_O_2_, 10 mM TM, 10 mM DDT (final concentrations). Fresh 5 × 5-mm hyphal plugs were inoculated on V8 medium plates. Colony diameters were measured by fine crosshair. Photographs were taken after 5 days. Then inhibition rate was calculated using the following formula: the inhibition rate = (growth rate on plates without stress – growth rate on plates with stress)/growth rate on plates without stress.

### Transformation of *P. sojae*

To generate the knockout mutants of *PsGRASP*, the CRISPR-mediated gene replacement approach (Fang and Tyler, 2016) was used. Briefly, the mCherry encoding gene with two 1.0-kb flanking fragments was used as the donor DNA in homology-directed repair (HDR). *P. sojae* was transformed using the PEG-mediated protoplast transformation protocol (Fang et al., 2017). Putative transformants were selected by growth on 10% (v/v) V8 medium containing 50 μg/mL geneticin (G418) and screened by PCR using primer combinations as shown in Supplementary Table 3. Primer set F1/R1 located within the *PsGRASP* ORF was used to screen for successful deletions of *PsGRASP*. Primer sets F2/R2 and F3/R3 were used to detect homologous recombination events. HDR events were analyzed by Sanger sequencing to confirm the clean replacement of *PsGRASP* by *mCherry*. To generate overexpression mutants of *PsGRASP*, the plasmid pTOR-GFP containing the *PsGRASP* gene and GFP fusion at its C-terminal was used for transformation.

### Mycelium Growth and Zoospore Production

All strains were cultured on 10% V8 juice agar medium at 25°C in the dark for growth rate measurements. Colony diameters were measured after 5 days and photographed.

To quantify sporangia and zoospore production, three round mycelial disks (6 mm in diameter) cut from the same culture were inoculated in 20 mL of 10% V8 broth and for 3 days in the dark. Sporangia or zoospores were prepared by repeatedly washing mycelia with sterile water every 30 min and incubating in 5 ml of water at 25°C for about 8 h until most mycelia developed sporangia. Sporangia were then gently mixed with a blender to obtain a homogenous mixture. Subsequently, three random 100 μL samples were taken to examine under a microscope examination to count the number of sporangia in each sample. The sporangia suspensions were transferred to 4°C for 1 and 2 h to allow zoospore release, respectively. We manually counted the number of zoospores in 50 μL of sporangia suspension and calculated the release rate of zoospore under microscope. All assays were repeated at least three times.

### RNA Extraction and Gene Expression Analysis

For qRT-PCR, samples at different asexual life stages, including vegetative hyphae, sporulating hyphae, zoospores, cysts, and germinated cysts, were collected as previously described (Ye et al., 2011; Sheng et al., 2015). Hyphae cultured in 10% V8 juice at 25°C in the dark for 3 days were washed with Plich liquid medium followed by incubation in 20 ml of Plich liquid medium. H_2_O_2_, DTT (5 mM) or TM (0.5 mM) were added to the Plich liquid medium. All samples were harvested at 5- and 10-min post incubation.

Total RNA samples were extracted using the RNA simple Total RNA Kit (Tiangen, China). For to reverse transcription, all RNA samples were DNase I-treated using a DNase kit, following the manufacturer’s protocol. First strand cDNA was synthesized from 1 μg of total RNA using oligo (dT) primers with an M-MLV reverse transcriptase kit (Vazyme, Cat. No.: R021-01), following the manufacturer’s protocol. SYBR green qRT-PCR assays were used to measure target gene expression with the aid of the primer pair listed in Supplementary Table 3. All reactions were performed on an ABI 7500 Fast Real-Time PCR System (Applied Biosystems, Inc., Foster City, CA, United States). The results were analyzed using ABI 7500 sequence detection software. qRT-PCR was performed as previously described with the *P. sojae* endogenous *ActinA* gene [Joint Genome Institute (JGI) GeneID: 108986] used as the reference gene (Sheng et al., 2015). The relative transcript accumulation levels were calculated based on the expression level in *P. sojae* (WT) mycelia whose value was set as 1.0. All assays were repeated at least three times.

### Fluorescence Microscopy

For fluorescence observation, the mycelia of PsGRASP-OE mutants were cultivated in 10% V8 juice medium for 2 days. Golgi Tracker Red dye was used to stain the mycelial of PsGRASP-OE mutants. Cells were visualized using confocal microscope (LSM 710; Carl Zeiss) at the specific excitation and emission wavelengths (GFP, 488 and 495–530 nm; and RFP, 561 and 575–640 nm).

### Virulence Assays

The soybean susceptible cultivar Hefeng 47 was grown in plastic pots containing vermiculite at 25°C in the dark for 4 days to acquire etiolated seedlings. Each seedling was directly inoculated with 10 μL of zoospore suspension (20 zoospores/μL) on the hypocotyl and maintained in 80% relative humidity in the dark at 25°C for 48 h before photographs were taken. Virulence was quantified by determining the ratio of *P. sojae* to soybean DNA in the infected soybean tissues as measured by qPCR.

To examine infectious hyphal expansion within the soybean tissue, infected epidermal cells were collected at 12 and 24 hour post infection (hpi) and soaked in Trypan Blue solution (10 ml H_2_O, 10 ml lactic acid, 10 ml glycerol, 10 g phenol, and 10 mg trypan blue) for 2 h. After destaining in chloral hydrate and water, the epidermis of the treated hypocotyls was examined using an Olympus 1 × 71 inverted microscope. Each strain was tested using at least two different preparations of zoospores and five plants. All assays were repeated at least three times.

To visualize the accumulation of H_2_O_2_ in the infected soybean tissue, the inoculated seedlings were collected at 24 hpi and soaked in 1 mg/ml DAB for 8 h in the dark. After destaining in an ethanol/acetic acid solution (47:1 [v/v]) for 4 h, the infected epidermis cells were examined by using an Olympus 1 × 71 inverted microscope.

### Laccase Activity Assay and the Quantification of Putative Extracellular Laccase-Encoding Genes

Laccase activity assay was performed following a previously reported procedure (Sheng et al., 2015). All strains were inoculated on LBA medium supplemented with 0.2 mM 2, 29 -azino-bis-3-ethylbenzothiazoline-6-sulfonic acid (ABTS, Sigma, United States) and incubated for 7 days at 25°C in the dark. The transcript accumulation levels of *PsLAC1-5* in mutants were compared with that in P6497 by qRT-PCR at mycelial stage. The *actin* gene from *P. sojae* was used as a constitutively expressed endogenous control and ΔΔCt method was used to calculate the relative transcription level. At least three biological replicates were performed for each data point.

## Results

### PsGRASP Is a Golgi Reassembly Stacking Protein Preferentially Expressed at the Infection Stages

**FIGURE 1:**
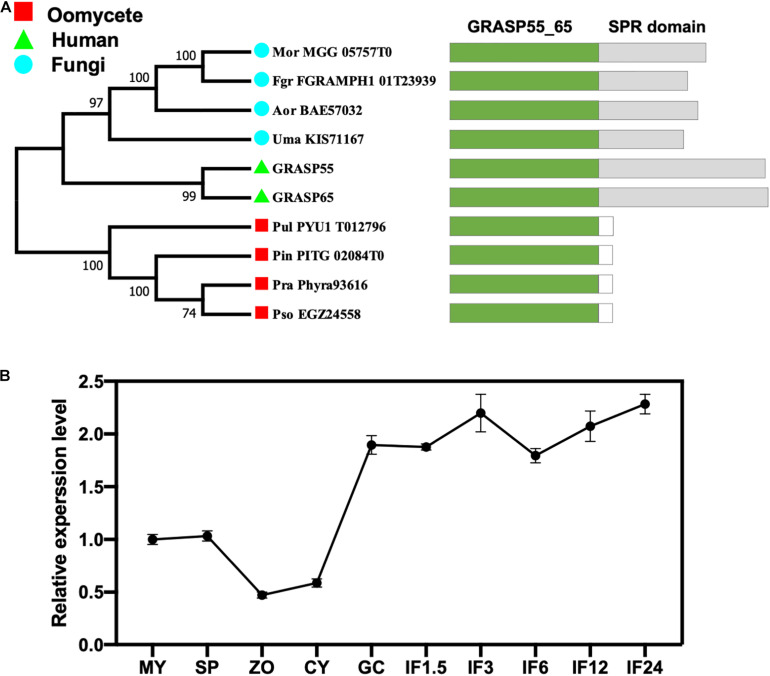
Identification of the GRASP protein (PsGRASP) in *P. sojae*. **(A)** The phylogenetic tree of ten predicted GRASPs from human, fungi, and oomycetes. They all have the conserved GRASP55_65 domain with the SPR domain missing in oomycete GRASPs. The phylogenetic tree was constructed with MEGA X using the maximum-likelihood method. Thousand bootstrap replicates were performed. Colored rectangles in the schematic diagrams of protein structures represent the different domains in each protein. **(B)** The transcript accumulation profile of *PsGRASP* in *P. sojae* life cycle and the infection stages, which was determined by qRT-PCR using RNAs extracted from vegetative mycelia (MY), sporangia (SP), zoospores (ZO), cysts (CY), germinated cysts (GC), and samples of different infection stages (IF 1.5, 3, 6, 12, and 24 refer to materials taken after the respective hours of inoculating hyphae on soybean leaves). Relative *PsGRASP* expression levels were calculated using the MY values as a reference.

All eukaryotic GRASPs feature an N-terminal GRASP55/65 PDZ-like domain with two PDZ-like motifs (Wang et al., 2005; Zhang and Wang, 2016). Conserved domain searches of the GRASP55/65 PDZ-like domain using the Pfam database identified a single GRASP candidate from each of the four oomycetes and four fungi species examined (Figure 1A). Phylogenetic analysis revealed that all four fungal GRASPs were grouped with human GRASP55 and GRASP65 in the same clade, while the four oomycete GRASPs form another clade (Figure 1A). Unlike fungal or human GRASPs, oomycete GRASPs do not have the serine proline-rich (SPR) domain in their C-terminals. The divergent evolution of oomycete and fungal/human GRASPs is consistent with their distant phylogeny at the kingdom level.

We named the 269-amino-acid GRASP identified in *P. sojae* PsGRASP (JGI protein ID: Physo3_485203), and examined its gene expression pattern in mycelial, sporangia, zoospores, cysts, germination cysts, and multiple infection stages (IF1.5, 3, 6, 12, and 24 hpi) via quantitative reverse-transcription PCR (qRT-PCR). Zoospores and cysts had the lowest *PsGRASP* transcript accumulation (Figure 1B). *PsGRASP* expression turned consistently higher in germinating cysts and the infection stages (Figure 1B). This expression pattern suggests that PsGRASP primarily functions during the infection stages of *P. sojae.*

### *PsGRASP*-Knockout Mutants Have Reduced Growth Rates

**FIGURE 2:**
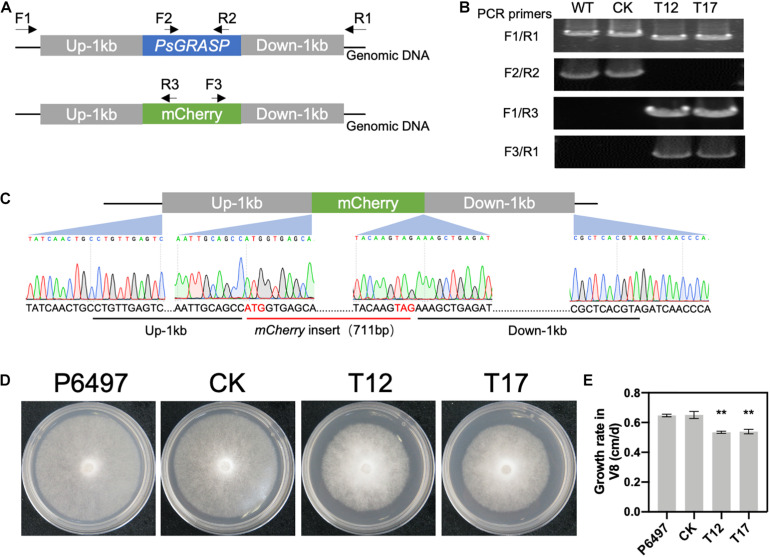
CRISPR-mediated gene replacement of *PsGRASP* and the colony morphology of *PsGRASP*-knockout mutants. **(A)** Locations of the primers used to screen the HDR mutants. **(B)** Analysis of genomic DNA from the wild-type (P6497), two *PsGRASP*-knockout mutants (T12 and T17), and an unsuccessful knockout line (CK) using the primers shown in **(A)**. **(C)** Sanger sequencing of the junction regions confirmed that the *PsGRASP* ORF has been precisely replaced by the mCherry gene. **(D)** Colonies of P6497, CK, T12, and T17 were cultured on 10% V8 agar medium at 25°C in the dark for 5 days. **(E)** the quantified growth rates of the indicated colonies from **(D)**. Each experiment was repeated three times. Scale bars denote SD. **P < 0.01, Student’s *t*-test.

*PsGRASP* knockout in the wild-type strain P6497 was achieved by replacing it with the mCherry gene using the CRISPR/Cas9 system (Figure 2A). Successful replacement of *PsGRASP* in two independent mutants (T12 and T17) were verified by PCR (Figure 2B) and sequencing (Figure 2C). In the growth rate assay, the colony sizes of both *PsGRASP*-knockout strains were significantly smaller than those of P6497 and a control strain (CK) with unsuccessful *PsGRASP* knockout (Figure 2D). Compared to P6497 and CK, both *PsGRASP*-knockout mutants showed approximately 16% lower mycelial growth rates (Figure 2E). The results suggest that PsGRASP is required for the normal growth of *P. sojae.*

### *PsGRASP* Deletion Affects Zoospore Release

**FIGURE 3:**
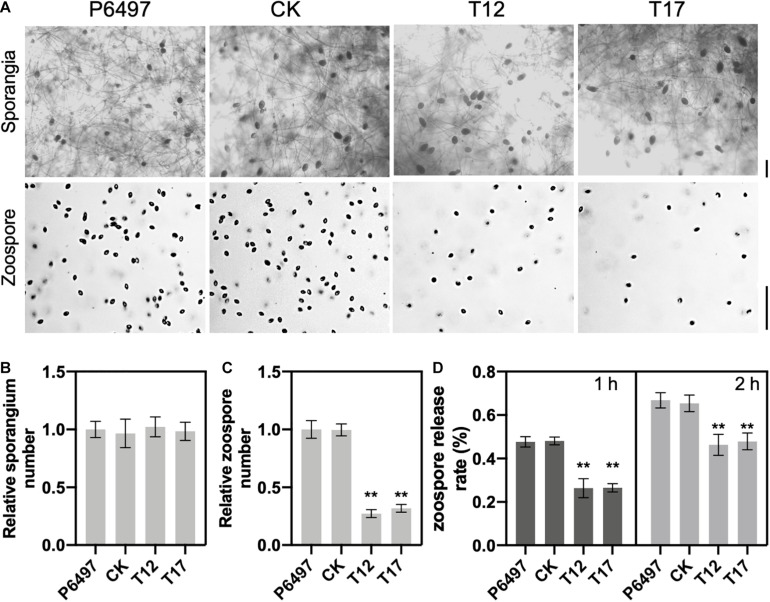
*PsGRASP* is required for zoospore release. **(A)** Microscopic visualization of sporangia and zoospores. Scale bar, 50 μm. **(B,C)** Quantification of zoospore and sporangia production. All experiments were repeated three times with similar results obtained. **(D)** Reduced numbers of zoospore production were observed in both *PsGRASP*-knockout mutants (T12 and T17). Zoospores released by the indicated lines were counted after 1 and 2 h of sporangial induction. The data shown represent three independent experiments. Error bars denote SD. **P < 0.01, Student’s *t*-test.

To determine the role of GRASP on spore production, *PsGRASP* knockout mutants T12 and T17, CK and P6497 were cultured in liquid V8 medium for 3 days, and then used to induce sporangium formation (Figure 3A). Zoospore production was examined 9 h after flushing the mycelia with water (Figure 3A). Although all four lines exhibited similar sporangium densities (Figure 3B), zoospore production was significantly reduced in T12 and T17 as compared to that of CK and P6497 (Figure 3C). Next, we assessed their zoospore release rates. Zoospore release was induced in sporangium suspensions by incubation at 25°C. Release rates were measured at 1 and 2 h after incubation. After 1 h of incubation, both T12 and T17 showed dramatically reduced zoospore release rates (around 25%) as compared to P6497 and CK, which both released more than 45% of their zoospores (Figure 3D). The differences were still significant after 2 h of incubation (Figure 3D). The results above suggest that PsGRASP is involved in zoospore release but not sporulation.

### PsGRASP Is Localized to Golgi and Induced by ER Stress

**FIGURE 4:**
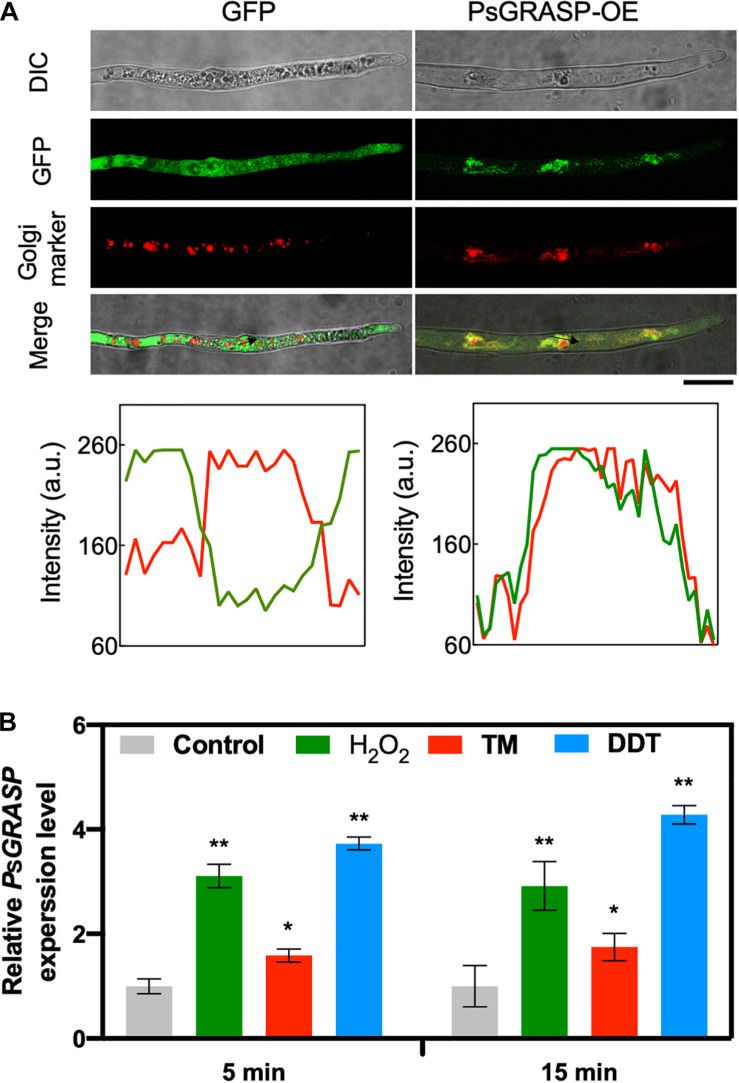
PsGRASP is localized to Golgi apparatus. **(A)** From top to bottom: co-localization of PsGRASP-OE (PsGRASP fused with a GFP tag) and Golgi-Tracker Red marker in *P. sojae*. Images of differential interference contrast (DIC), GFP, and red fluorescence derived from the Golgi-Track Red dye. Merge overlay of DIC, GFP fluorescence, and Golgi-Track Red dye staining. Bar, 20 μm. Relative fluorescence intensity along with the block arrows in **(A)**. Lines and graph analysis of the co-localization patterns. Green line: GFP or PsGRASP-OE. Red line: Golgi (stained with the Golgi-Track Red dye). **(B)** Expression profiles of *PsGRASP* under different stress conditions. The wild-type mycelium was subject to treatments with control (H_2_O), H_2_O_2_, TM or DTT. The control value (1.0) was set as a reference. Results represent mean values ± SD from three independent experiments. *P < 0.05, **P < 0.01, Student’s *t*-test.

To determine the subcellular localization of PsGRASP, a green fluorescent protein (GFP) tag was fused to its C-terminal (PsGRASP-OE) and used for *P. sojae* transformation. The GFP-only vector was used as a control. GFP signals generated by PsGRASP-OE were visible in punctate structures throughout the hyphae (Figure 4A). The Golgi apparatus was visualized using the Golgi Tracker Red dye in the hyphae, the colocalization of PsGRASP-OE was shown in merge (yellow) indicated that PsGRASP and Golgi markers nearly overlapped (Figure 4A) and PsGRASP-OE and Golgi Tracker signals were well overlapped with a Pearson’s co-localization value of 0.74 ± 0.01 (Figure 4A). In contrast, the GFP-only signals were not colocalized with the Golgi location marker (Figure 4A). The colocalization patterns were confirmed through lines and graph analysis (Figure 4A). These results indicated that PsGRASP is likely to be located to Golgi in *P. sojae*.

PsGRASP transcript accumulation levels increased by about one to three folds after 5-min treatments of H_2_O_2_ or the ER stress inducers tunicamycin (TM) and dithiothreitol (DTT) (Figure 4B). The increases were largely stable with prolonged treatment time of 15 min (Figure 4B). These observations suggest that *PsGRASP* expression responds rapidly to oxidative and ER stress.

### PsGRASP Is Essential for Oxidative and ER Stress Tolerance

**FIGURE 5:**
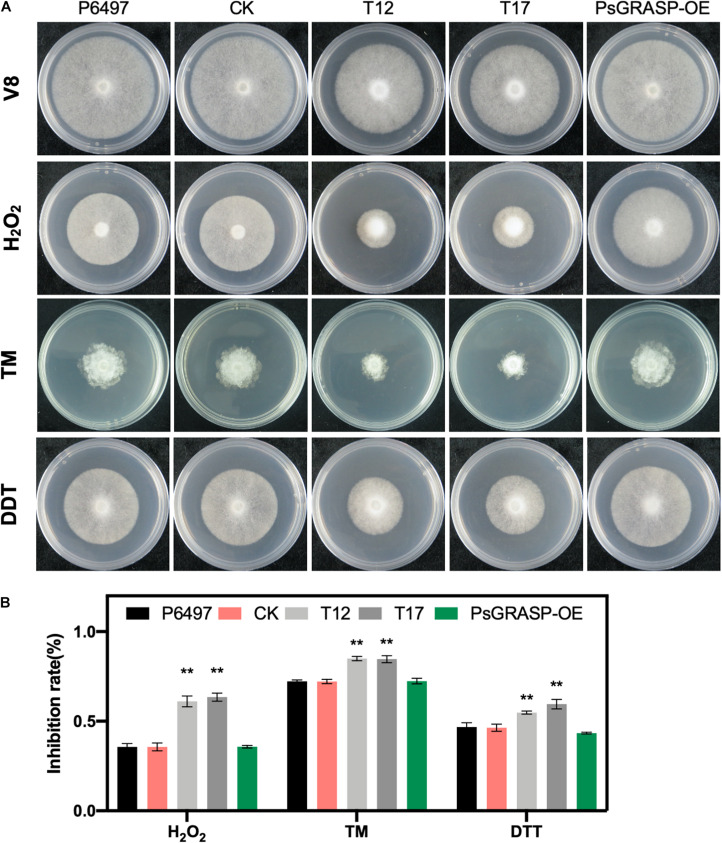
Mycelial growth assays under different stress conditions. **(A)** Mycelial growth assays of P6497, CK, T12, T17, and PsGRASP-OE lines on V8 agar medium and media supplemented with H_2_O_2_, TM or DTT. TM, tunicamycin. DTT, Dithiothreitol. **(B)** Colony diameters were measured after five and a half days of growth. Growth inhibition Rates were calculated for each treatment relative to the growth on the V8 agar medium without supplements. Three biological replicates were performed. Error bars denote SD. **P < 0.01, Student’s *t*-test.

To further investigate the function of PsGRASP in oxidative and ER stress, we cultivated T12, T17, P6497, CK, and PsGRASP-OE lines on V8 medium supplemented with H_2_O_2_, TM, or DTT (Figure 5A). P6497, CK, and PsGRASP-OE showed similar levels of responses to all three oxidative and ER stress inducers (Figure 5B). In contrast, both T12 and T17 were more sensitive to these inducers and exhibited significantly higher growth inhibition rates (Figure 5B). These results indicate that PsGRASP is required for *P. sojae* to cope with oxidative and ER stress.

### PsGRASP Is Required for Pathogenicity

**FIGURE 6:**
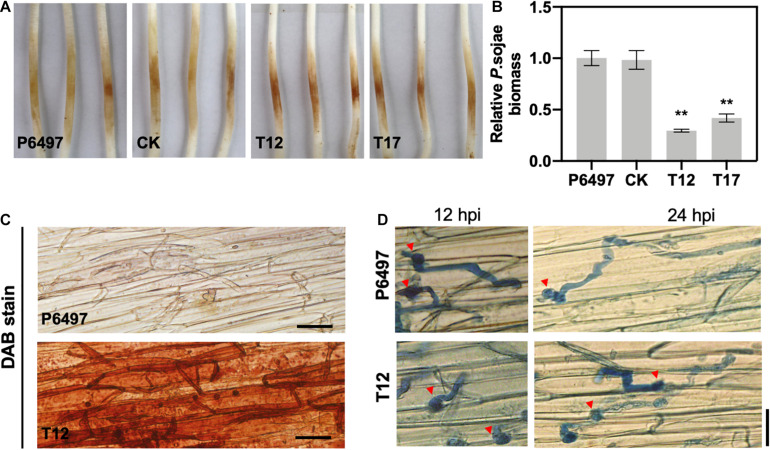
The *PsGRASP* knockout mutants are impaired in virulence. **(A)** Lesions on soybean (cultivar Hefeng 47) etiolated hypocotyls at 48 hours post-inoculation (hpi) of P6497, CK, T12, or T17 zoospores. Experiments were repeated three times with similar results. **(B)** Relative pathogen biomass in inoculated etiolated hypocotyls, which is expressed as the ratio between the amounts of *P. sojae* and soybean DNA detected at 48 hpi. The ratio of P6497/soybean was set at 1.0. Error bars denote SD. **P < 0.01, Student’s *t*-test. **(C)** DAB staining of the hypocotyls epidermis infected by P6497 and T12. **(D)** Microscopic observations of invasive hyphae in the epidermis of soybean hypocotyls at 24 hpi. Trypan Blue staining was performed on the epidermis of seedling hypocotyls. Red arrowheads indicate cysts. Bar, 20 μm.

Virulence assays were performed to explore the role of PsGRASP in pathogenicity. Zoospores of T12, T17, P6497, and CK were inoculated on 4-day-old etiolated seedlings of Hefeng 47, which is a *P. sojae*-susceptible soybean cultivar. Symptoms were examined 48 h post-inoculation. Seedlings inoculated with *PsGRASP*-knockout strains (T12 or T17) exhibited significantly reduced lesions when compared to those inoculated with P6497 or CK (Figure 6A). Measurements of relative *P. sojae* biomass at the inoculation sites confirmed that the pathogen biomass decreased by approximately 60–70% in the infected tissues of T12 or T17 (Figure 6B). These results suggest that PsGRASP is required for *P. sojae* virulence at the infection stages.

To explore the underlying mechanism of PsGRASP-mediated virulence, we monitored host-derived H_2_O_2_levels via 3,3’-diaminobenzidine (DAB) staining. At 24 hpi, only weak H_2_O_2_ accumulation was detected in soybean epidermal cells inoculated with P6497, whereas T12-inoculated soybean exhibited strong H_2_O_2_ accumulation (Figure 6C). The result showed that the *PsGRASP*-knockout strain is incapable of scavenging host-derived ROS. When the infected tissues were stained with Trypan Blue and examined under a microscope for more details, P6497 was able to penetrate the epidermal cell and expand the invasive hyphae to adjacent cells at 12 hpi, whereas the hyphae of T12 was restricted to the regions surrounding the penetration site. At 24 hpi, the infectious hyphae of the P6497 were much more abundant, which had extended into neighboring cells while the expansion of hyphal was severely retarded in T12 (Figure 6D). Taken together these results indicated the PsGRASP has a role in the pathogenicity of *P. sojae*.

### PsGRASP Deletion Leads to the Decrease of Extracellular Laccase Activity

**FIGURE 7:**
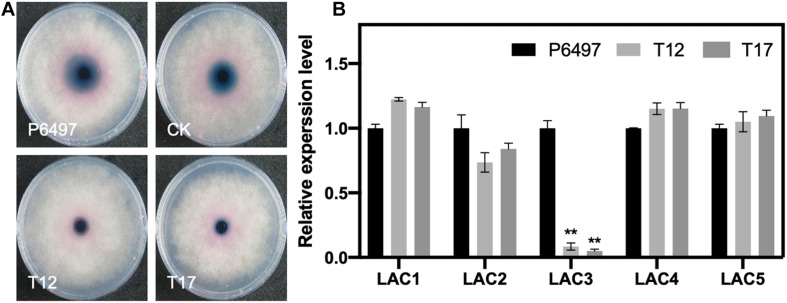
Detection of laccase activity and the relative expression of putative laccase-encoding genes in the *PsGRASP*-knockout mutants. **(A)** Mycelial mats of P6497, CK, T12, and T17 were inoculated on LBA media containing 0.2 mM ABTS and grown for 7 days. The oxidized ABTS (dark purple) was measured as an index of laccase activity. **(B)** qRT-PCR analysis of five highly expressed laccase-encoding genes (*PsLAC1-5*) in P6497, T12, and T17. Three biological replicates were performed. Error bars denote SD. **P < 0.01, Student’s *t*-test.

Since GRASPs are required for unconventional secretion and the secretion of virulence effectors (Giuliani et al., 2011; Kmetzsch et al., 2011), we propose that PsGRASP may regulate the activity or expression of certain *P. sojae* extracellular proteins. Extracellular laccase activity has been reported to contribute to oomycete or fungal pathogenicity (Mayer and Staples, 2002; Sheng et al., 2015). Thus, we employed a 2,2 0 -azino-bis (3-ethylbenzothiazoline-6 sulfonic acid) (ABTS)-based oxidation assay to compare the extracellular laccase activity of P6497, CK, T12, and T17. Compared to P6497 and CK, T12 and T17 accumulated significantly reduced amount of oxidized ABTS as revealed by much smaller scale of dark purple staining underneath the mycelial mat (Figure 7A). The result demonstrates that *PsGRASP*-knockout strains have reduced extracellular laccase activity.

It has been reported that *P. sojae* has 15 putative laccase-encoding genes (*PsLACs*) and five of them (*PsLAC1-5*) show high-level expression during the life cycle (Sheng et al., 2015). Our secretion signal peptide (SP) prediction result (Supplementary Table 1) showed that 11 out of 15 PsLACs contain a SP. Two PsLACs were identified as unconventional secretion proteins. They also showed relatively low transcript accumulation levels. To explore whether the reduction of laccase activity in *PsGRASP*-knockout strains was due to transcriptional suppression of the five highly expressed *PsLACs*, we performed qRT-PCR assays to examine the expression of *PsLAC1-5* genes in P6497, T12 and T17. While no significant difference was detected for the expression of *PsLAC1-3* and *5* among the three lines, *PsLAC3* expression decreased by 80–90% in T12 and T17 (Figure 7B). As a laccase-encoding gene being upregulated at GC and early infection stages, the active of laccase encoded by *PsLAC3* is likely to be suppression target of PsGRASP.

## Discussion

Golgi reassembly stacking proteins are conserved in eukaryotes with functions in Golgi structure formation, protein trafficking, and cell migration. Although the importance of GRASPs in fungi virulence has been highlighted in some reports, little is known about their roles in oomycete pathogens. In this study, we predicted a single GRASP protein from each of the four representative oomycetes and then focused on the functional analysis of PsGRASP in the major soybean pathogen *P. sojae*. Assays using *PsGRASP*-knockout lines demonstrate its essential roles in vegetable growth, asexual development, stress responses and pathogenicity.

Although GRASP orthologs and paralogs have been identified extensively in yeast, parasites, flies and mammals, they are absent in plants (Zhang and Wang, 2020). However, the plant-infecting oomycete pathogens examined all have a single copy of *GRASP* gene in their genomes. Unlike their human or fungal homologs, oomycete GRASPs have a relatively independent evolution theme featuring the missing of the SPR domain. This feature can also found in the yeast pathogen *C. neoformans* (Kmetzsch et al., 2011). The SPR domain contains multiple phosphorylation sites, whose phosphorylation inhibit GRASP oligomerization in mitosis and perhaps also in stress and pathological conditions (Wang et al., 2005; Tang et al., 2012; Joshi et al., 2015). Thus, oomycete GRASPs might evolve a novel regulation mechanism different from human and fungal.

The observation that *PsGRASP* expression was up-regulated specifically at the infection stages (Figure 1B) suggests its important role in pathogenicity. Given that Golgi formation depends on GRASPs in mammalian cells (Xiang and Wang, 2010; Bekier et al., 2017), PsGRASP may also play a role in Golgi formation in *P. sojae*. The reduced mycelial growth and zoospore release rates observed in *PsGRASP*-knockout lines (Figures 2, 3) could be at least partially resulted from Golgi malfunctions.

Several recent reports link certain transcription factors and mitogen-activated protein kinases (MAPKs) to *Phytophthora* growth and zoospore production (Li et al., 2014; Jiang et al., 2018; Lin et al., 2018; Judelson and Ah-Fong, 2019). For example, the SLT2-type PsMPK1 is required for *P. sojae* normal growth and zoosporogenesis (Li et al., 2014, p. 1). As an essential event occurred in *P. sojae* development and infection, autophagy can be induced during sporangium formation and cyst germination, with multiple autophagy-related gene upregulated at the zoospore stage (Chen et al., 2017). Given that GRASPs are phosphorylation substrates of MAPKs and serve as membrane tethers to facilitate autophagosome-lysosome fusion (Jesch et al., 2001; Zhang and Wang, 2020), we hypothesize that PsGRASP and PsMPK1 may coordinately regulate autophagy, growth, sporangium formation, and zoosporogenesis in *P. sojae*.

Although most GRASPs are Golgi-localized, a subset of these proteins can also be detected on other membrane organelles. For example, the single Drosophila GRASP ortholog dGRASP is also found on the ER exit sites (ERES) and on ER itself (Schotman et al., 2008). The additional ER-localization of GRASPs is consistent with their critical role in ER stress response. GRASP-depleted cells under ER stress exhibit abnormal Golgi including fragmentation, shorter cisternae, and fewer cisternae per stack (Bekier et al., 2017; Zhang and Wang, 2020). In our study, we observed that PsGRASP was colocalized with Golgi marker, and suggest that PsGRASP is localized to Golgi apparatus, where it may act (Figure 4). *PsGRASP* transcripts accumulate rapidly in response to oxidative and ER stress, and consistently, *PsGRASP*-knockout mutants exhibited increased sensitivity to H_2_O_2_ and two ER stress inducers DTT and TM. These results clearly point to a positive role of PsGRASP in *P. sojae* tolerance to oxidative and ER stress.

Plant-derived reactive oxygen species (ROS) accumulation at the pathogen penetration site is considered to be one of the earliest responses for pathogen-associated molecular pattern (PAMP)-triggered immunity mechanisms in plants (Torres and Dangl, 2005; Jones and Dangl, 2006). Plant pathogens including *Phytophthora* have developed several strategies to counteract ROS toxicity, survive from ROS in harsh environments, and invade host cells successfully. For example, PsHSF1 is a transcription factor that detoxifies the plant oxidative burst, which is critical for pathogenicity in *P. sojae* (Sheng et al., 2015). Pathogens also secrete effectors targeting plant immunity system to attenuate pattern-triggered immunity (PTI) responses including ROS generation (He et al., 2020). In our study, *PsGRASP*-knockout mutants have hypersensitivity to H_2_O_2_, limited infectious hyphae growth and reduced virulence in susceptible soybean plants (Figure 6). We therefore propose that the phenotypes described above result from the loss or severe weakening of the ability to adapted host-derived H_2_O_2_ in the knockout mutants.

In most plant pathogens, secreted peroxidases are important for plant-derived ROS scavenging during plant–pathogen interactions (Judelson and Ah-Fong, 2019). Given that laccase is an oxidase that has been widely identified in plant pathogens as an important virulence factor (Sheng et al., 2015; Staerck et al., 2017), we analyzed the extracellular laccase activity and the expression of five highly expressed laccase-encoding genes (*PsLAC1-5*) in *PsGRASP*-knockout mutants. Compared to the wild type, both mutants have much lower extracellular laccase activity with *PsLAC3* expression being dramatically downregulated (Figure 7). PsLAC3 is likely to be a direct activation target in PsGRASP-mediated ROS scavenging.

Golgi reassembly stacking proteins are required for unconventional secretion in several organisms (Kinseth et al., 2007; Duran et al., 2010; Manjithaya et al., 2010). Since the two predicted PsLACs encoding unconventional secretion laccases have extremely low expression levels, their potential roles in pathogenicity and the involvement of PsGRASP remain unclear. Nonetheless, GRASP is required for normal polysaccharide secretion and microbial virulence (Kmetzsch et al., 2011), and a key regulator of vesicular export of RNA in *C. neoformans* (Peres da Silva et al., 2018). Large number of effector proteins were secreted by *Phytophthora* to destroy the immunity of host. Effectors are discharged from pathogens through at least two mechanisms. Most of effectors have a classic SP and that thought to be secreted via the classic Golgi-mediated pathway (Judelson and Ah-Fong, 2019). Furthermore, the unconventional secretion proteins have been documented in *Phytophthora* (Liu et al., 2014). To demonstrated the role of PsGRASP in protein secretion in *P. sojae*, secretomes were prepared form P6497 and the PsGRASP knockout mutant T12 by label-free quantification. Total of 301 differently expressed proteins (DEPs) that containing 91 unconventional secretion proteins and 210 classic secretion proteins were identified between P6497 and T12 (Supplementary Figure 1). Gene ontology classification analysis showed that much more biological process categories were abundant in the down-regulation expressed proteins. Furthermore, we found the secretion of an acyl-coenzyme A-binding protein (ACBP, Ps112733) was affected in T12 which has report in other species, like *D. discoideum* (Kinseth et al., 2007). Taken together, the secretomes analysis provide a potential role of PsGRASP in secretion regulation. However, there are still many unanswered questions about the secretion mechanism of effector proteins and whether PsGRASP involved to regulate the secretion of these effectors.

Overall, we identified and functionally characterized a novel gene *PsGRASP*, which encodes a Golgi reassembly stacking protein in *P. sojae*. PsGRASP is essential for vegetative growth, ER stress response, virulence and pathogenicity. Extracellular laccase activity is one of the downstream mechanisms regulated by PsGRASP. In the future, additional biochemical and molecular pathways linked to pathogenicity, GRASP and Golgi-mediated/unconventional protein secretion are expected to be uncovered in oomycetes and other plant pathogens. The above results will enrich our knowledge of oomycete pathogenesis, secretion pathway of effectors, and consequently provide potential targets for oomycete pathogen control.

## Data Availability Statement

The original contributions presented in the study are included in the article/Supplementary Material, further inquiries can be directed to the corresponding author/s.

## Author Contributions

DD conceived and designed the research. XZ and DS collected the data and completed the bioinformatics analyses. JS, YP, PJ, RX, and HQ performed the experiments. JS, HP, and DD wrote the manuscript. All the authors read and approved the final manuscript.

## Conflict of Interest

The authors declare that the research was conducted in the absence of any commercial or financial relationships that could be construed as a potential conflict of interest.

## Footnotes

1 www.cbs.dtu.dk/services/SignalP-3.0

2 http://www.cbs.dtu.dk/services/SecretomeP

## Supplementary Material

Supplementary Figure 1 Differentially expressed proteins between the secretomes of P6497 and PsGRASP knockout mutant T12. **(A)** Electrophoretic profile on SDS-PAGE and concentration of each protein protocol for P6497 and T12. M, protein ladder molecular weight. **(B)** The number of differentially expressed proteins (DEPs) between P6497 and T12. Proteins were predicted the signal peptide (SP). **(C)** Gene Ontology functional classification of the down-regulation expressed proteins.

Supplementary Table 1 Expression profiles and secretion predictions of putative laccase-encoding genes in *P. sojae*.

Supplementary Table 2 Information about protein identification by label-free quantification.

Supplementary Table 3 Primers used in this study.

## References

[B1] Behnia R. Barr F. A. Flanagan J. J. Barlowe C. Munro S. (2007). The yeast orthologue of GRASP65 forms a complex with a coiled-coil protein that contributes to ER to Golgi traffic. *J. Cell Biol.* 176 255–261. 10.1083/jcb.200607151 17261844 PMC2063951

[B2] Bekier M. E. Wang L. Li J. Huang H. Tang D. Zhang X. (2017). Knockout of the Golgi stacking proteins GRASP55 and GRASP65 impairs Golgi structure and function. *Mol. Biol. Cell* 28 2833–2842. 10.1091/mbc.e17-02-0112 28814501 PMC5638586

[B3] Chen L. Zhang X. Wang W. Geng X. Shi Y. Na R. (2017). Network and role analysis of autophagy in *Phytophthora sojae*. *Sci. Rep.* 7:1879. 10.1038/s41598-017-01988-7 28500315 PMC5431975

[B4] Duran J. M. Anjard C. Stefan C. Loomis W. F. Malhotra V. (2010). Unconventional secretion of Acb1 is mediated by autophagosomes. *J. Cell Biol.* 188 527–536. 10.1083/jcb.200911154 20156967 PMC2828925

[B5] Erwin D. Ribeiro O. (1996). *Phytophthora Diseases Worldwide*. St. Paul, Minn.: APS Press.

[B6] Fang Y. Tyler B. M. (2016). Efficient disruption and replacement of an effector gene in the oomycete Phytophthora sojae using CRISPR/Cas9. *Mol. Plant Pathol.* 17 127–139. 10.1111/mpp.12318 26507366 PMC6638440

[B7] Fang Y. Cui L. Gu B. Arredondo F. Tyler B. M. (2017). Efficient Genome Editing in the Oomycete *Phytophthora sojae* Using CRISPR/Cas9. *Curr. Protc. Microbiol.* 44:25. 10.1002/cpmc.25 28166383

[B8] Gee H. Y. Noh S. H. Tang B. L. Kim K. H. Lee M. G. (2011). Rescue of ΔF508-CFTR Trafficking via a GRASP-Dependent Unconventional Secretion Pathway. *Cell* 146 746–760. 10.1016/j.cell.2011.07.021 21884936

[B9] Gisi U. Sierotzki H. (2015). “Oomycete Fungicides: Phenylamides, Quinone Outside Inhibitors, and Carboxylic Acid Amides,” in *Fungicide Resistance in Plant Pathogens*, eds Ishii H. Hollomon D. W. (Tokyo: Springer Japan), 145–174. 10.1007/978-4-431-55642-8_10

[B10] Giuliani F. Grieve A. Rabouille C. (2011). Unconventional secretion: a stress on GRASP. *Curr. Opin. Cell Biol.* 23 498–504. 10.1016/j.ceb.2011.04.005 21571519

[B11] He Q. McLellan H. Boevink P. C. Birch P. R. J. (2020). All Roads Lead to Susceptibility: The Many Modes of Action of Fungal and Oomycete Intracellular Effectors. *Plant Commun.* 1:100050. 10.1016/j.xplc.2020.100050 33367246 PMC7748000

[B12] Jesch S. A. Lewis T. S. Ahn N. G. Linstedt A. D. (2001). Mitotic Phosphorylation of Golgi Reassembly Stacking Protein 55 by Mitogen-activated Protein Kinase ERK2. *Mol. Biol. Cell* 12 1811–1817. 11408587 10.1091/mbc.12.6.1811 PMC37343

[B13] Jiang L. Situ J. Deng Y. Z. Wan L. Xu D. Chen Y. (2018). PlMAPK10, a Mitogen-Activated Protein Kinase (MAPK) in Peronophythora litchii, Is Required for Mycelial Growth, Sporulation, Laccase Activity, and Plant Infection. *Front. Microbiol.* 9:426. 10.3389/fmicb.2018.00426 29568294 PMC5852060

[B14] Jones J. D. G. Dangl J. L. (2006). The plant immune system. *Nature* 444 323–329. 10.1038/nature05286 17108957

[B15] Joshi G. Bekier M. E. Wang Y. (2015). Golgi fragmentation in Alzheimer’s disease. *Front. Neurosci.* 9:340. 10.3389/fnins.2015.00340 26441511 PMC4585163

[B16] Judelson H. S. Ah-Fong A. M. V. (2019). Exchanges at the Plant-Oomycete Interface That Influence Disease. *Plant Physiol.* 179 1198–1211. 10.1104/pp.18.00979 30538168 PMC6446794

[B17] Judelson H. S. Blanco F. A. (2005). The spores of *Phytophthora*: weapons of the plant destroyer. *Nat. Rev. Microbiol.* 3 47–58. 10.1038/nrmicro1064 15608699

[B18] Kinseth M. A. Anjard C. Fuller D. Guizzunti G. Loomis W. F. Malhotra V. (2007). The golgi-associated protein GRASP is required for unconventional protein secretion during development. *Cell* 130 524–534. 10.1016/j.cell.2007.06.029 17655921

[B19] Kmetzsch L. Joffe L. S. Staats C. C. de Oliveira D. L. Fonseca F. L. Cordero R. J. B. (2011). Role for Golgi reassembly and stacking protein (GRASP) in polysaccharide secretion and fungal virulence. *Mol. Microbiol.* 81 206–218. 10.1111/j.1365-2958.2011.07686.x 21542865 PMC3124575

[B20] Li A. Zhang M. Wang Y. Li D. Liu X. Tao K. (2014). PsMPK1, an SLT2-type mitogen-activated protein kinase, is required for hyphal growth, zoosporogenesis, cell wall integrity, and pathogenicity in *Phytophthora sojae*. *Fungal Genet. Biol.* 65 14–24. 10.1016/j.fgb.2014.01.003 24480463

[B21] Lin L. Ye W. Wu J. Xuan M. Li Y. Gao J. (2018). The MADS-box Transcription Factor PsMAD1 Is Involved in Zoosporogenesis and Pathogenesis of *Phytophthora sojae*. *Front. Microbiol.* 9:2259. 10.3389/fmicb.2018.02259 30319576 PMC6165875

[B22] Liu T. Song T. Zhang X. Yuan H. Su L. Li W. (2014). Unconventionally secreted effectors of two filamentous pathogens target plant salicylate biosynthesis. *Nat. Commun.* 5:4686. 10.1038/ncomms5686 25156390 PMC4348438

[B23] Manjithaya R. Anjard C. Loomis W. F. Subramani S. (2010). Unconventional secretion of Pichia pastoris Acb1 is dependent on GRASP protein, peroxisomal functions, and autophagosome formation. *J. Cell Biol.* 188 537–546. 10.1083/jcb.200911149 20156962 PMC2828923

[B24] Mayer A. M. Staples R. C. (2002). Laccase: new functions for an old enzyme. *Phytochemistry* 60 551–565. 10.1016/s0031-9422(02)00171-1 12126701

[B25] Peres da Silva R. Martins S. T. Rizzo J. Dos Reis F. C. G. Joffe L. S. Vainstein M. (2018). Golgi Reassembly and Stacking Protein (GRASP) Participates in Vesicle-Mediated RNA Export in Cryptococcus Neoformans. *Genes* 9:genes9080400. 10.3390/genes9080400 30096850 PMC6115741

[B26] Rabouille C. Linstedt A. D. (2016). GRASP: A Multitasking Tether. *Front. Cell Dev. Biol.* 4:1. 10.3389/fcell.2016.00001 26858948 PMC4726779

[B27] Schotman H. Karhinen L. Rabouille C. (2008). dGRASP-mediated noncanonical integrin secretion is required for Drosophila epithelial remodeling. *Dev. Cell* 14 171–182. 10.1016/j.devcel.2007.12.006 18267086

[B28] Shemorry A. Harnoss J. M. Guttman O. Marsters S. A. Kõmûves L. G. Lawrence D. A. (2019). Caspase-mediated cleavage of IRE1 controls apoptotic cell commitment during endoplasmic reticulum stress. *eLife* 8:47084. 10.7554/eLife.47084 31453810 PMC6711704

[B29] Sheng Y. Wang Y. Meijer H. J. Yang X. Hua C. Ye W. (2015). The heat shock transcription factor PsHSF1 of *Phytophthora sojae* is required for oxidative stress tolerance and detoxifying the plant oxidative burst. *Environ. Microbiol.* 17 1351–1364. 10.1111/1462-2920.12609 25156425

[B30] Staerck C. Gastebois A. Vandeputte P. Calenda A. Larcher G. Gillmann L. (2017). Microbial antioxidant defense enzymes. *Microbial Pathog.* 110 56–65. 10.1016/j.micpath.2017.06.015 28629723

[B31] Tang D. Yuan H. Wang Y. (2010). The Role of GRASP65 in Golgi Cisternal Stacking and Cell Cycle Progression. *Traffic* 11 827–842. 10.1111/j.1600-0854.2010.01055.x 20214750 PMC3278861

[B32] Tang D. Yuan H. Vielemeyer O. Perez F. Wang Y. (2012). Sequential phosphorylation of GRASP65 during mitotic Golgi disassembly. *Biol. Open* 1 1204–1214. 10.1242/bio.20122659 23259055 PMC3522882

[B33] Torres M. A. Dangl J. L. (2005). Functions of the respiratory burst oxidase in biotic interactions, abiotic stress and development. *Curr. Opin. Plant Biol.* 8 397–403. 10.1016/j.pbi.2005.05.014 15939662

[B34] Tyler B. M. Tripathy S. Zhang X. Dehal P. Jiang R. H. Aerts A. (2006). *Phytophthora* genome sequences uncover evolutionary origins and mechanisms of pathogenesis. *Science* 313 1261–1266. 10.1126/science.1128796 16946064

[B35] van Ziel A. M. Largo-Barrientos P. Wolzak K. Verhage M. Scheper W. (2019). Unconventional secretion factor GRASP55 is increased by pharmacological unfolded protein response inducers in neurons. *Sci. Rep.* 9:1567. 10.1038/s41598-018-38146-6 30733486 PMC6367349

[B36] Vinke F. P. Grieve A. G. Rabouille C. (2011). The multiple facets of the Golgi reassembly stacking proteins. *Biochem. J.* 433 423–433. 10.1042/BJ20101540 21235525

[B37] Wang S. Welsh L. Thorpe P. Whisson S. C. Boevink P. C. Birch P. R. J. (2018). The Phytophthora infestans Haustorium Is a Site for Secretion of Diverse Classes of Infection-Associated Proteins. *Mbio* 9 e1216–e1218. 10.1128/mBio.01216-18 30154258 PMC6113627

[B38] Wang Y. Z. Satoh A. Warren G. (2005). Mapping the functional domains of the Golgi stacking factor GRASP65. *J. Biol. Chem.* 280 4921–4928. 10.1074/jbc.M412407200 15576368 PMC4443495

[B39] Xiang Y. Wang Y. (2010). GRASP55 and GRASP65 play complementary and essential roles in Golgi cisternal stacking. *J. Cell Biol.* 188 237–251. 10.1083/jcb.200907132 20083603 PMC2812519

[B40] Ye W. Wang X. Tao K. Lu Y. Dai T. Dong S. (2011). Digital gene expression profiling of the Phytophthora sojae transcriptome. *Mol. Plant. Microbe. Interact.* 24 1530–1539. 10.1094/MPMI-05-11-0106 21848399

[B41] Yelinek J. T. He C. Y. Warren G. (2009). Ultrastructural Study of Golgi Duplication in Trypanosoma brucei. *Traffic* 10 300–306. 10.1111/j.1600-0854.2008.00873.x 19207482

[B42] Zhang X. Wang Y. (2016). GRASPs in Golgi Structure and Function. *Front. Cell Dev. Biol.* 3:00084. 10.3389/fcell.2015.00084 26779480 PMC4701983

[B43] Zhang X. Wang Y. (2020). Nonredundant Roles of GRASP55 and GRASP65 in the Golgi Apparatus and Beyond. *Trends Biochem. Sci.* 45 1065–1079. 10.1016/j.tibs.2020.08.001 32893104 PMC7641999

