# Supplementary figures and images for: PsGRASP, a Golgi Reassembly Stacking Protein in Phytophthora sojae, Is Required for Mycelial Growth, Stress Responses, and Plant Infection
Source: Front Microbiol. 2021 Jul 8;12:702632. doi: 10.3389/fmicb.2021.702632 (PMC8297711; doi:10.3389/fmicb.2021.702632)

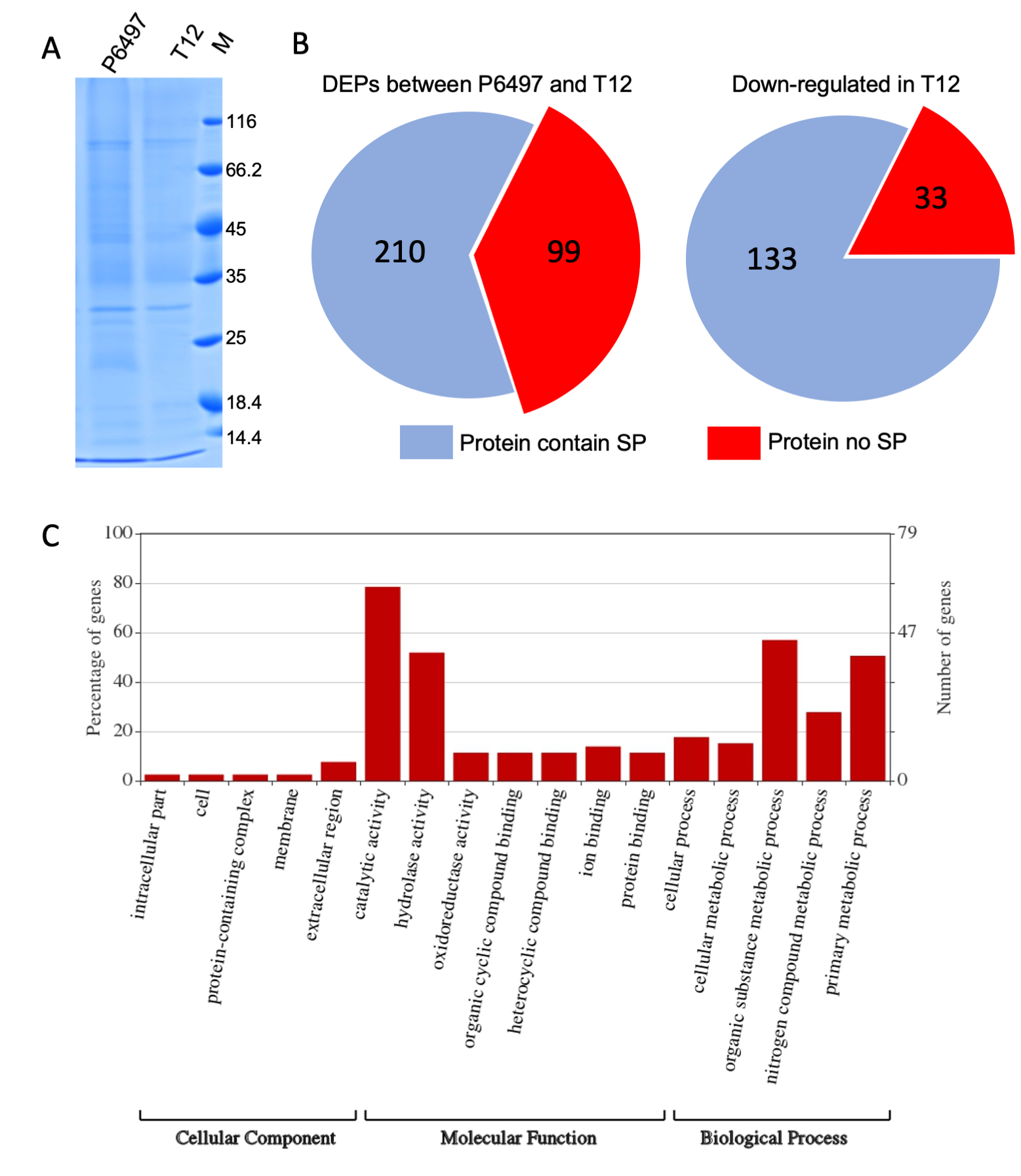

Supplement: Supplementary Figure 1 — Differentially expressed proteins between the secretomes of P6497 and PsGRASP knockout mutant T12. (A) Electrophoretic profile on SDS-PAGE and concentration of each protein protocol for P6497 and T12. M, protein ladder molecular weight. (B) The number of differentially expressed proteins (DEPs) between P6497 and T12. Proteins were predicted the signal peptide (SP). (C) Gene Ontology functional classification of the down-regulation expressed proteins. [file Image_1.tiff]
